# Interaction of Human Serum Album and C_60_ Aggregates in Solution

**DOI:** 10.3390/ijms12084964

**Published:** 2011-08-04

**Authors:** Maoyong Song, Shufang Liu, Junfa Yin, Hailin Wang

**Affiliations:** State Key Laboratory of Environmental Chemistry and Ecotoxicology, Research Center for Eco-Environmental Sciences, Chinese Academy of Sciences, Beijing 10085, China; E-Mails: smsong@rcees.ac.cn (M.S.); liushufang405@163.com (S.L.); jfyin@rcees.ac.cn (J.Y.)

**Keywords:** nC_60_, HSA, interaction, protein, fluorescence

## Abstract

An important property of C_60_ in aquatic ecotoxicology is that it can form stable aggregates with nanoscale dimensions, namely nC_60_. Aggregation allows fullerenes to remain suspended for a long time, and the reactivity of individual C_60_ is substantially altered in this aggregate form. Herein, we investigated the interaction of nC_60_ and human serum album (HSA) using the methods of fluorescence, fluorescence dynamics, circular dichroism (CD), and site marker competitive experiments. We proposed a binding model consistent with the available experimental results for the interactions of nC_60_ with HSA. During the interaction process, the structure and conformation of HSA were affected, leading to functional changes of drug binding sites of HSA.

## 1. Introduction

Since the discovery of fullerene (C_60_) in 1985 [1], concerns have been raised about the proposed applications of C_60_ and its derivatives in biology and pharmacology [2,3]. These potential applications include items as varied as antiviral, anticancer, or antioxidant agents [4], and drug delivery vehicles [5,6]. Despite the broad range of research focused on the application potential of fullerenes, their toxicological and environmental effects are still not well known [7]. With an octanol-water partition coefficient log K_OW_ of 6.67 and a solubility of less than 10^−9^ mg/L, pristine fullerene is poorly soluble in water [8]. For C_60_ fullerene, its interaction with proteins has been poorly studied due in particular to its low solubility in water, and hence much research has focused on the interactions between its water-soluble derivatives and some proteins such as HIV protease [9,10], a fullerene-specific antibody [11], human serum albumin (HSA) [12] and bovine serum albumin (BSA) [13].

An important property of C_60_ in aquatic ecotoxicology is its acquisition of charge and ability to form relatively stable clusters (referred to as nC_60_) in aqueous systems, by means of natural processes like water flow and mixing, as well as by vigorous stirring in the laboratory [14–16]. Aggregation allows fullerenes to remain suspended for weeks or months [17], this aspect of nC_60_ may provide a more complete understanding of how it will behave in natural systems or in organisms.

In previous work, BSA was found to adopt a more flexible conformational state on the boundary surface of gold nanoparticles as a result of the conformational changes in the bioconjugates [18]. It was also demonstrated that C_60_ nanoparticles can be stabilized by nonspecific adsorption of HSA and remain well dispersed even in the physiological environment [19]. Therefore, for further understanding of the transportation of nC_60_ fullerene in the bodies and the resulting related bio-effects, the interaction of nC_60_ fullerene with proteins is crucial and must be ascertained. In this study, the interaction between human serum albumin and nC_60_ in aqueous solution was investigated by fluorescence, fluorescence dynamics, circular dichroism (CD), and binding site marker competitive experiments.

## 2. Results and Discussion

### 2.1 The Fluorescence Quenching of HSA by nC_60_

**Figure 1.**
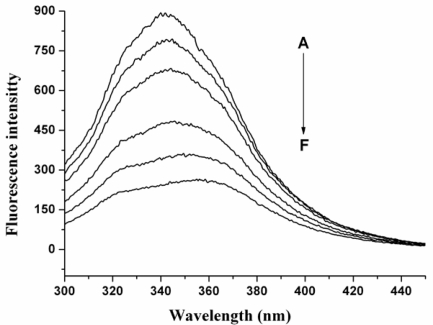
Fluorescence spectra of human serum album (HSA) in the presence of nC_60_ aggregates. HSA concentration was 20 μM. The concentration of C_60_ (from A to F) was 0, 1.41, 2.83, 5.66, 8.48, and 11.30 μM, respectively (pH = 7.4; ex = 280 nm).

Quenching refers to any process which decreases the fluorescence intensity of a given substance. A variety of processes can result in quenching, such as excited state reactions, energy transfer, complex-formation and collisional quenching. Figure 1 shows the fluorescence spectra of HSA in the absence and the presence of nC_60_ in phosphate buffer with an excitation wavelength of 280 nm. HSA displayed the maximum emission at a wavelength of 343 nm. The fluorescence intensity of HSA decreased with increasing concentration of nC_60_ and the maximum emission wavelength was slightly red shifted. It is known that the shift of maximum emission wavelength corresponded to a polarity change around the chromophore residues. Red shift indicates that the microenvironment of fluorophors in HSA is changed after addition of nC_60_.

### 2.2. Quenching Mechanism of nC_60_ to HSA

For dynamic quenching, the quenching is an additional process that deactivates the excited state besides radiative emission through the collision between the quencher and fluorophore. As for HSA, the dependence of the emission intensity on quencher concentration [*Q*] is given by the Stern-Volmer equation:

(1) $$F_{0}/F=1+K_{q}\tau_{0}[Q]=1+K_{SV}[Q]$$

where *F* and *F_0_* are the fluorescence intensity in the absence and in the presence of quencher, respectively; τ_0_ is the lifetime of HSA; *K_q_* is the bimolecular rate constant for the dynamic reaction of the quencher with the fluorophore; K_sv_ is called the Stern-Volmer constant.

For static quenching, the binding of a quencher (*Q*) to a protein (*M*) can be described with the following reaction:

$$\mathrm{nQ}+M↔{\mathrm{MQ}}_{n}$$

Then the binding constant (*K*) is given by

(2) $$K=\frac{\left[{\mathrm{MQ}}_{n}\right]}{[M]{\left[Q\right]}^{n}}$$

The relation between the fluorescence intensity and concentration of quencher [ *Q* ] is given by

(3) $${\left[M\right]}_{0}=[M]+[{\mathrm{MQ}}_{n}]$$

(4) $$\frac{F_{0}-F}{F}=\frac{{\left[M\right]}_{0}-[M]}{\left[M\right]}=\frac{\left[{\mathrm{MQ}}_{n}\right]}{\left[M\right]}=K{\left[Q\right]}^{n}$$

Rearrange Equation (4)

(5) $$\frac{F_{0}}{F}=1+K{\left[Q\right]}^{n}$$

**Figure 2.**
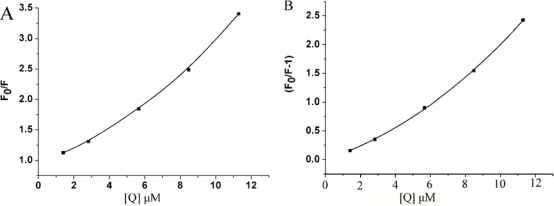
(**A**) The Stern-Volmer plot for the fluorescence quenching of HSA by nC_60_; (**B**) Nonlinear fitting analysis performed by plotting (*F_0_/F-1*) against [Q].

The quenching data were presented as a plot of $\frac{F_{0}}{F}$ *versus* [*Q*], and the Stern-Volmer plot is an upward curvature, concave towards the y-axis, as shown in Figure 2A.

This result indicates that HSA can be quenched both by collisions and by complex formation with C_60_ nanoparticles. Then the fractional fluorescence remaining $\left(\frac{F_{0}}{F}\right)$ is given by

(6) $$\frac{F}{F_{0}}=f(1+K_{\mathrm{SV}}[Q])$$

where *f* is the fraction not complexed, and (1 + *K_SV_*[*Q*]) is the fraction not quenched by collisional encounters.

According to the definition, *f* can be obtained from Equation (5), and then rearrangement of Equation (6) yields:

(7) $$(\frac{F_{0}}{F}-1)=K_{\mathrm{SV}}[Q]+K{\left[Q\right]}^{n}+K_{\mathrm{SV}}K{\left[Q\right]}^{n+1}$$

The nonlinear fitting analysis was performed by plotting $\left(\frac{F_{0}}{F}-1\right)$ against *Q* (Figure 2B). From the Stern-Volmer plot, the value of *K*_SV_ was 6.56 × 10^4^ L·mol^−1^. The *K_q_* was easily calculated according to Equation (1), 6.56 × 10^12^ L·mol^−1^·s^−1^, which is much higher than the maximal collided dynamic quenching constant (2.0 × 10^10^ L·mol^−1^·s^−1^) [20]. This result indicates that the fluorescence quenching of HSA by the addition of nC_60_ is mainly caused by static quenching. There is non-linearity obtained from Stern-Volmer when the nC_60_ concentration is lower than 11.30 μM. When nC_60_ concentration is higher than 11.30 μM, the intrinsic fluorescence of HSA is significantly quenched. The plot appears to be an upward curvature with increasing nC_60_ concentration, which is a characteristic feature of mixed quenching. This suggests that it is not a single quenching mechanism that exists in the binding process. The mechanism of HSA quenching caused by water-soluble pristine nC_60_ is different to that caused by water-soluble nC_60_ derivative [12].

### 2.3. The Conformation Study of HSA

**Figure 3.**
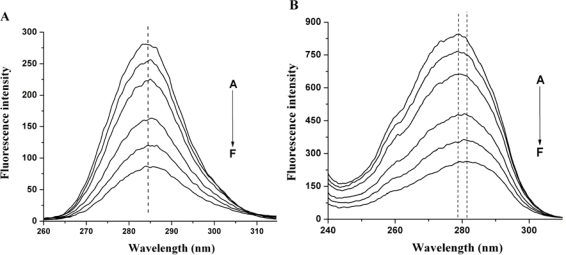
Synchronous fluorescence spectra of HSA in the presence of nC_60_. (**A**) Δλ = 15 nm; (**B**) Δλ = 60 nm. HSA concentration is 20 μM. The concentration of C_60_ (from A to F) was 0, 1.41, 2.83, 5.66, 8.48, 11.30 μM, respectively.

Synchronous fluorescence spectroscopy is a common method used to provide information about conformational changes of protein since the possible shift of maximum emission wavelength is related to the polarity of the environment. The synchronous fluorescence spectra of HSA-nC_60_ system are shown in Figure 3. The maximum emission wavelength of tyrosine residues has a small red shift (from 283 nm to 285 nm) when Δλ = 15 nm, indicating that there are some changes in the environment of the tyrosine residues. The maximum emission wavelength of tryptophan residues red shifts from 279.5 nm to 281.5 nm when Δλ = 60 nm (Figure 3B). This suggests that there is a less hydrophobic or more polar environment change around the tyrosine residues and tryptophan residues. This may be ascribed to the fact that the hydrophobic amino acid structure surrounding tryptophan residues in HSA tends to collapse slightly, resulting in tyrosine residues and tryptophan residues being more exposed to the aqueous phase.

**Figure 4.**
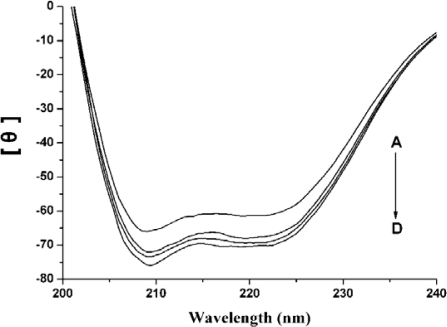
The circular dichroism (CD) spectra of the HSA-nC_60_ system. Concentration of HSA was 7.3 μM; Concentration of nC_60_ (from A to D) was 0, 3.38, 5.63, 11.8 μM, respectively.

Circular dichroism spectra can sensitively monitor conformation changes in the protein upon interaction with the ligand. In this experiment, the calculated α-helicity content of native HSA solution is 54.9%, and with the addition of nC_60_ to the native HSA solution, the α-helicity content of HSA increased to 59.8%, 61.2% and 62.0% (Figure 4). Apparently, the higher the added nC_60_ concentration, the more the α-helicity content. The increase of α-helicity content indicated that the binding of HSA and nC_60_ induces protein folding, which results in some hydrophobic regions becoming more compact.

### 2.4. Interaction between HSA and nC_60_

**Figure 5.**
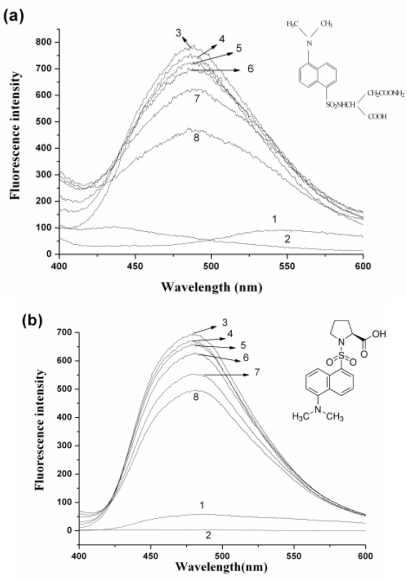
Effect of nC_60_ on binding site probe-HSA system. Concentrations of HSA and probes (DNSA and DP) were 20 μM. (**a**) 1: HSA only; 2: DNSA only; Ex = 350 nm; (**b**) 1: HSA only, 2: DP only; ex = 375 nm. Concentrations of C_60_ (from 3 to 8) were 0, 1.41, 2.83, 5.66, 8.48, 11.30 μM, respectively. The inserts correspond to the molecular structure of binding site probes.

It is well known that HSA has two major drug binding sites, site I and site II, which are located in the hydrophobic pocket of sub-domain IIA and sub-domain IIIA, respectively. In order to identify the nC_60_-binding site on HSA, two probes were used. One probe of HSA is dansylamide (DNSA), whose binding site is located in the region of sub-domain IIA (sudlow site I); another probe, dansylproline (DP) is bound to HSA in the sub-domain IIIA (sudlow site II) [21,22]. During the experiment, nC_60_ was gradually added to the solution of HSA with site probes held in equimolar concentrations (20 μM). No absorption of these two probes to nanoparticles of C_60_ was observed in capillary electrophoresis analysis (data not shown). As shown in Figure 5, only the probes or HSA alone have no fluorescence signal within the wavelengths 400–600 nm. When the probes and HSA are mixed together, the DNSA-HSA complex has a maximum emission wavelength at 487 nm (excitation wavelength 350 nm) and DP-HSA has a maximum emission wavelength at 480 nm (excitation wavelength 375 nm). With the addition of nC_60_ to the HSA-probe solution, the fluorescence intensity of the HSA-probe complex was significantly lower than that without nC_60_, indicating that the binding of DNSA/DP to HSA was affected by adding nC_60_. The fluorescence intensity of DNSA-HSA was lower than that of DP-HSA, indicating that nC_60_ could more strongly influence the binding of DNSA to HSA than that of NP to HSA.

### 2.5. Representation of Interaction of HSA and nC_60_

**Figure 6.**
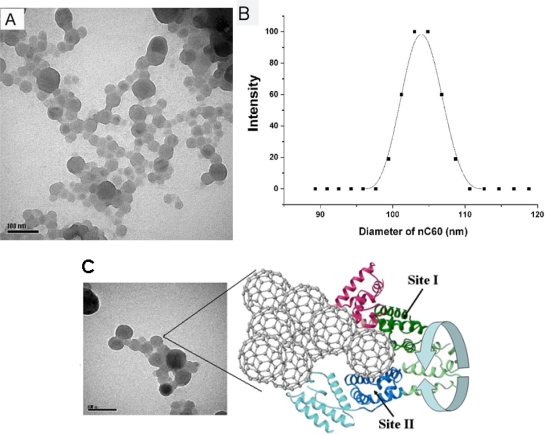
(**A**) Transmission electron microscopy (TEM) image of nC_60_; (**B**) The mean hydrodynamic diameter of the nC_60_ measured by dynamic light scattering (DLS); (**C**) Schematic representation of HSA interacting with aggregate nC_60_ surface.

Our results above indicate that the fluorescence quenching and conformational changes of HSA can be attributed to the interaction between HSA and nC_60_. C_60_ fullerene has also been reported to be bound to the subdomain IIA of HSA using time-resolved fluorescence decay experiments, docking calculations, and binding site alignment methods [13,23]. Here we have employed several approaches in order to address the issue of fullerene interactions with proteins. What needs to be especially pointed out is that it is difficult to incorporate nC_60_ into the binding pocket because the size of nC_60_ is larger than protein molecules. The hydrodynamic diameter of the nC_60_ nanoparticles was determined to be from 50 nm to 110 nm by TEM and DLS, respectively (Figure 6A, B). Ganazzoli *et al.* reported atomistic computer simulations of some albumin subdomains on a hydrophobic graphite surface [24]. According to these simulations, we proposed a binding model to describe the interaction between HSA and nC_60_ (Figure 6C). In this model, HSA is adsorbed on the surface of nC_60_ aggregate which leads to the fluorescence quenching and conformational changes of HSA. The interaction sites were presumed to be near to Site I and Site II of HSA; following the structure and conformation of HSA was affected during adsorption. In our previous study, we have observed that HSA could compete for the surface of nC_60_ with functional enzymes [25].

## 3. Experimental

### 3.1. Materials

C_60_ (99.5% purity) was obtained from Sigma Chemical Company, St. Louis, USA. HSA (98% purity, essentially fatty acid and globulin free) was purchased from NEB (USA). All solutions were prepared in ultrapure water with the resistivity of 18.2 MΩ·cm (ALGA system, UK). Phosphate buffer (10 mM, pH = 7.4) was prepared with chemicals of analytical pure grade, obtained from commercial sources, and filtered with a 0.22-μmm filter (Millipore, Bedford, MA, USA). All other materials were of analytical pure grade.

### 3.2. Preparation and Characterization of Water-Soluble nC_60_

C_60_ suspensions were prepared according to the method described previously [26]. Ten milligrams of C_60_ was completely dissolved in 10 mL toluene. Then 2.5 mL C_60_ toluene solution was added into 50 mL water. After ultrasonic treatment for approximately 4 h, toluene was allowed to evaporate under vacuum for at least 1 h. The mean hydrodynamic diameter of the nC_60_ nanoparticles was determined by transmission electron microscopy (TEM) and dynamic light scattering (DLS) (BI-20OSM laser light scattering spectrometer, Brookhaven, USA).

### 3.3. Methods

Absorption spectra were recorded on a UV-2102PCS UV-Vis spectrophotometer (UNICO, USA) equipped with a 1.0 cm quartz cuvette at room temperature. The wavelength range was recorded from 200 to 500 nm.

All fluorescence emission spectra were recorded in the range of 300–450 nm on a LS-55 spectrofluorimeter (PE, USA) equipped with 100 μL quartz cells. The bandwidth of excitation and emission (3 nm) and scan speed (300 nm/min) were kept constant within each data set. Fluorescence intensity at 343 nm was used for calculation in Stern-Volmer equation. All measurements were performed at room temperature (∼25 °C).

Binding site marker competitive experiments were carried out by the fluorescence titration methods. The concentration of HSA and dansylamide/dansylproline (DNSA/DP) were all kept at 10 μM. Then nC_60_ was gradually titrated into the HSA-DNSA/DP systems. Fluorescence spectra were recorded with an excitation wavelength of 370 nm (for DNSA) and 375 nm (for DNP) in the range of 400–600 nm.

Circular dichroism (CD) spectroscopy was performed on a J-810 spectropolarimeter (Jasco Co., Japan) over a wavelength of 200–260 nm. The scanning speed was set at 500 nm/min. Each spectrum was the average of three successive scans and appropriate buffer solutions running under the same conditions were taken as blank and their contributions were subtracted from the experimental spectra.

## 4. Conclusions

We observed a quenching of fluorescence of HSA in the presence of nC_60_, and fluorescence quenching mechanism was investigated by the Stern-Volmer equation which showed a characteristic feature for the combined fluorescence quenching. The binding of HSA to nC_60_ increases the polar environment of the Trp residue, resulting in a red shift in the fluorescence spectra. The CD results showed that the percentage of α-helicity of HSA increased, revealing that the protein becomes more compact upon association with nC_60_. The results indicate that the fluorescence quenching and conformational changes of HSA can be attributed to the interaction between HSA and nC_60_. In the binding site marker competitive experiment, the binding of DNSA/DP to HSA was affected by adding nC_60_. We propose that HSA interacted with aggregate nC_60_ surface, and led to the functional changes of drug binding sites of HSA.
